# Lysine-urethane-based tissue adhesion for mastectomy—an approach to reducing the seroma rate?

**DOI:** 10.1007/s00404-020-05801-1

**Published:** 2020-11-04

**Authors:** B. Boeer, J. Schneider, B. Schoenfisch, C. Röhm, S. Paepke, E. Oberlechner, R. Ohlinger, A. Hartkopf, S. Y. Brucker, M. Hahn, M. Marx

**Affiliations:** 1 Department of Women’s Health, University Hospital of Tuebingen, Calwerstraße 7, 72076 Tuebingen, Germany; 2 Department of Urology, Klinikum Am Steinenberg, Reutlingen, Germany; 3 Research Institute for Women’s Health, University of Tuebingen, Tuebingen, Germany; 4 Department of Obstetrics and Gynaecology, Technical University of Munich, Munich, Germany; 5 Department of Gynaecology and Obstetrics, Ernst-Moritz-Arndt University Greifswald, Greifswald, Germany; 6 Department of Plastic, Reconstructive and Breast Surgery, Elblandklinikum Radebeul, Radebeul, Germany

**Keywords:** TissuGlu^®^, Breast cancer therapy, Seroma formation, Drainage, Surgical adhesive

## Abstract

**Purpose:**

Postoperative seromas are a problem in the surgical treatment of breast cancer. The aim of the study was to evaluate whether the lysine-urethane-based tissue adhesive TissuGlu^®^ without drainage is equal/ non-inferior to standard mastecomy with drainage.

**Methods:**

The study was designed as a prospective, randomized, multicentre non-inferiority study comparing the use of TissuGlu^®^ without drainage with standard wound care with a drain insertion in ablative breast procedures. The number of clinical interventions, quality of life and wound complications were followed-up for 90 days in both groups.

**Results:**

Although the statistical power was not reached, twice as many clinical interventions were performed in the TissuGlu^®^ group than in the drainage group, especially aspirations of clinically relevant seromas (*p* = 0.014). The TissuGlu^®^ group produced overall less wound fluid, but developed a clinically relevant seroma (100% vs. 63%) which made an intervention necessary. Less hospitalisation time was observed in the TissuGlu^®^ group, but the complication rate was higher. There was no significant difference in regards to postoperative pain. In summary the non-inferiority of TissuGlu^®^ compared to standard drainage couldn’t be reached.

**Discussion:**

The present evaluation shows no advantage of the tissue adhesive TissuGlu^®^ in terms of seroma formation and frequency of intervention compared to a standard drainage for mastectomies, but the shorter inpatient stay certainly has a positive effect on the quality of life.

## Introduction

Seromas are the most frequent complication in the surgical treatment of breast cancer, particularly occurring in ablative procedures. Since breast cancer is the most common cancer in women in western industrialised countries and still cannot be treated with breast-conserving therapy in about 30% of cases, seromas are seen in breast clinics on a daily basis. According to the literature, seromas occur in 9.1–92% of ablative procedures [1]. Seroma is defined as an abnormal accumulation of endogenous, serous and in the later course lymphatic fluid between the ventral and dorsal surfaces of the wound cavity. It occurs mainly during surgery in which tissue is resected over a large area and a free space (dead space) is created. If the quantity of serum secreted is reabsorbed by the surrounding tissue, a seroma remains clinically asymptomatic. In most cases, it only becomes a clinically relevant problem with potential delay of further treatment as a result of secondary complications such as infections or secondary wound healing disorders caused by separation of the dorsal from the ventral surface of the wound cavity [1]. A variety of techniques for reducing seromas have been described in the literature—all with moderate success [2]. Closed multi-channel suction drainage has so far established itself as the standard for wound care [3, 4]. However, this can be accompanied by pain, an increase in hospitalization time [5] or reduced well-being with an increased level of anxiety [6] and represents a potential entry point for pathogens with subsequent infection [7].

Publications from the field of abdominoplasty have shown that lysine-urethane-based tissue adhesives—such as TissuGlu^®^ tissue adhesive—have the potential to minimize the occurrence of seroma formation [8–10]. Promising experiences from previous case reports and small case–control studies with the use of TissuGlu^®^ tissue adhesive in breast surgery formed the basis for the present study [11–14].

The present study is based on a prospective, randomized, multicentre non-inferiority study [15], which aimed to compare the use of TissuGlu^®^ without drainage with standard wound care with a drain insertion in ablative breast procedures. Seven study centres in Germany participated. Since the study was discontinued by the sponsor, only the cases from Tuebingen will be used.

## Materials and methods

The study design (Pro-100–0132, 181/2016 MPG 23, Ethics Committee) was to include 42 mastectomies per study arm. The test group was to be treated with TissuGlu^®^ without drains for wound closure, the control group with drains as a standard comparison.

The number of postoperative clinical interventions was chosen as the primary endpoint with the aim of demonstrating the hypothetical non-inferiority of TissuGlu^®^ application without a drain system. Clinical intervention was defined as drain removal and any invasive manipulative measure (Table 2).

Secondary endpoints were seroma formation, the cumulative drain-, aspiration- and total volume, the number of days of treatment and the number of days until drain removal. Other secondary endpoints were assessment of postoperative quality of life in terms of pain, sleep and mobility using a non-standardized questionnaire which was completed by the patient at every postoperative visit and at defined times after discharge.

**Fig. 1:**
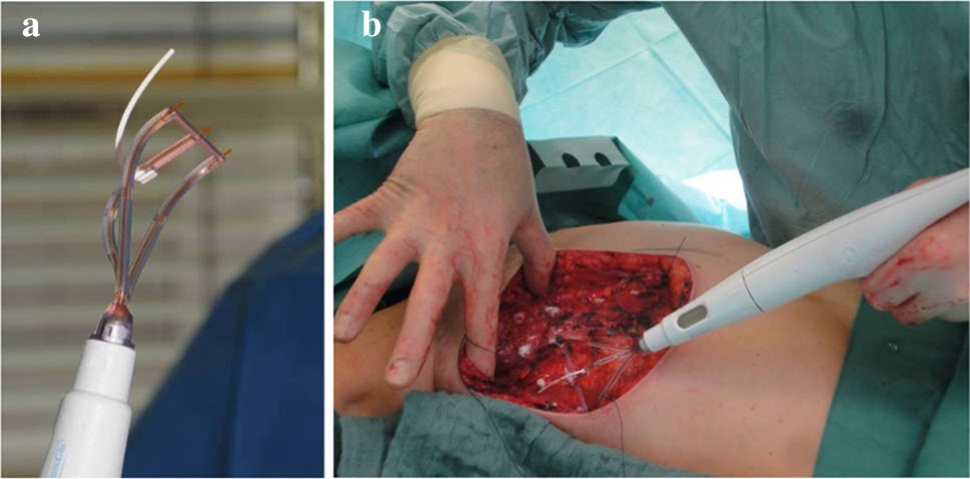
Illustration of the TissuGlu^®^ Applicator (**a**), and the application of TissuGlu^®^ in situ (**b**)

Randomisation was stratified by centre. Enrolled patients were randomized in the operating theatre after induction of anaesthesia. The centres were provided with numbered and sealed envelopes with the randomisation codes. Depending on randomisation, either a multi-channel suction drain was inserted after the mastectomy or TissuGlu^®^ was used to seal the surface and close the dead space without a drain. TissuGlu^®^ was applied drop by drop in a standardized manner using an applicator which delivers 3 drops of adhesive, each with a volume of 0.025–0.040 ml, in a line spaced 2.5 cm apart (Fig. 1).

The total volume applied related to the wound area to be sealed and was measured and documented for each patient. A pressure bandage was then applied to the area in all patients for 24 h. All operations were performed by senior surgeons certified by OnkoZert. The weight of the ablated material and the number of lymph nodes removed were recorded.

In the control group with a drain, output was documented over 24 h and the drain was removed if the secretion was below 30 ml/24 h on two consecutive days.

**Fig. 2:**
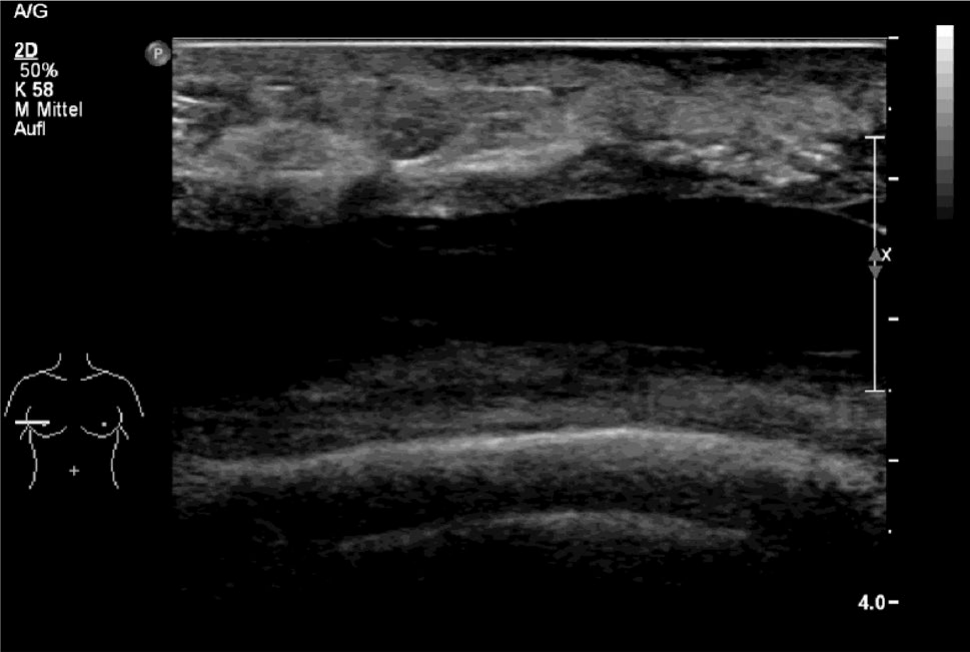
Illustration of clinically relevant seroma formation (> 1 cm) in the ultrasound of a patient of the TissuGlu^®^ group

Post-operative follow-up was performed daily as an in-patient (daily FU) and on days 7, 14, 30, 60 and 90 after discharge. Follow-up checks included anamnesis, local wound inspection and quality of life assessment. Wound inspection was based on medical inspection and palpation and signs of infection, suture dehiscence or haematomas were documented. If a seroma was suspected on palpation or if symptoms such as pain, tension, discomfort and local redness were reported, an ultrasound scan was performed. If the ultrasound scan showed a seroma depth of more than 1 cm, i.e. a clinically relevant seroma, aspiration was performed (Fig. 2). The aspirated volume was documented.

Pain was assessed using a 100 mm visual-analogue scale for the operated side (0 mm = none to 100 mm = maximum pain). The subjective restriction of mobility and sleep was documented as a numerical value (1 = none to 10 = maximum restriction). The questionnaire also contained specific questions concerning negative factors influencing mobility and sleep. In addition to postoperative pain, patients were asked about restrictions due to drain-associated factors such as the collection device or the drain insertion site, as well as restrictions because of seroma aspiration. Multiple answers were possible.

The sponsor terminated the study prematurely. The main reason for termination was the complex follow-up scheme with slow recruitment because, apart from the Tuebingen study centre, no other centre was able to meet the expected recruitment rate.

This publication presents data from 14 patients who were enrolled at the Tuebingen centre between February and October 2016. The patients underwent either a simple mastectomy or a mastectomy with sentinel node biopsy (SLNB).

8 patients in the intervention group were treated with intraoperative application of TissuGlu^®^. In the control group, 6 patients underwent breast removal with drain insertion. Since one patient underwent a bilateral mastectomy, a total of 7 treatments in the control group could be evaluated, and data from a total of 15 treatments is therefore available.

### Statistical analysis

Data was collected and analysed with SPSS Version 24. Since the study was terminated early and sample size was small, analysis was not possible as planned. Continous data was described by means and standard deviations (SD) and differences between TissuGlue^®^ and control group were assessed by the Wilcoxon-Mann–Whitney rank test. In case of ties being a problem, we used wilcox_test() from package coin in R version 3.5.1 to calculate the p-value. Nominal data was characterized by numbers and percentages and differences between the two groups were compared using Fisher’ exact test. To describe the repeated measurements of the variable pain, a linear regression model with the factors time and group was formulated and the individuals were modelled as a random factor. Goodness-of-fit was assessed by Nakagawa‘s Pseudo *R*^2^. For all tests, a significance level of 5% was chosen.

## Results

### General characteristics

The mean values in the intervention and control groups did not differ significantly with regard to age (mean 60.4 years, SD 14.7 vs. mean 62.0 years, SD 11.5; *p* = 0.852), BMI (mean 28.9 kg/m^2^, SD 4.9 vs. mean 24.4 kg/m^2^, SD 2.3; *p* = 0.108), weight of ablated tissue (mean 602 g, SD 332 vs. mean 426 g, SD 157; *p* = 0.232) and the number of lymph nodes removed (3.1, SD 3.1 vs. 3.7, SD 3.1; *p* = 0.613) (Table 1).

**Table 1:** Patient characteristics

Patient collective Tuebingen	TissuGlu^®^ number | percentage respective mean (SD)	Control number | percentage respective mean (SD)
Patients	8	6
Breast removal	8	7*
Gender (w/m)	7 / 1	6 / 0
Age [years]	60.4 (14.7)	62.0 (11.5)
BMI [kg/m^2^]	28.9 (4.9)	24.4 (2.3)
Mastectomy	2 | 18%	0 | 0%
Mastectomy + SLNB (*n* | %)	6 | 72%	7 | 100%
Weight of ablated tissue [g]	602 (332)	426 (157)
Number of lymph nodes removed	3.1 (3.1)	3.7 (3.1)
History of smoking		
Never smoked	5 | 63%	3 | 50%
Active smoker	3 | 38%	2 | 33%
Stopped smoking	0 | 0%	1 | 17%
Pre-treatment		
Chemotherapy	2 | 25%	0 | 0%
Radiotherapy	2 | 25%	2 | 33%
Antihormonal therapy	1 | 13%	2 | 33%

* one patient with bilateral mastectomy

Three patients in the TissuGlu^®^ group had already received the following treatments several years before: Two patients had undergone ipsilateral breast conservation surgery (BCS) + radiotherapy. One of these patients received adjuvant chemotherapy, the other received adjuvant antihormonal therapy. Another patient underwent a contralateral modified radical mastectomy and received adjuvant chemotherapy.

In the control group, two patients had also previously undergone BCS + radiotherapy, one on the ipsilateral side (initial diagnosis 1994), the other on the contralateral side (initial diagnosis 2008). One of these patients also received anti-hormonal therapy. Furthermore, the control group included one patient with contralateral mastectomy and subsequent antihormonal therapy (initial diagnosis 2011).

### Primary endpoint

In the TissuGlu^®^ group, a total of 44 clinical interventions (66% of all interventions) were performed on 5 of 7 operated breasts; in the control group, 22 (33%) clinical interventions were performed on 8 of 8 operated breasts (Table 2).

**Table 2:** Number of clinical interventions

Type of clinical intervention	TissuGlu^®^ number | percentage	Control number | percentage
Needle aspiration	43 | 98%	12 | 55%
Surgical intervention	1 | 2%	0 | 0%
Postoperative drain insertion	0 | 0%	1 | 5%
Drain intervention	0 | 0%	1 | 5%
Drain removal	0 | 0%	8 | 35%
Total	44 | 100%	22 | 100%

In addition to the aspirations performed in both groups (55 | 83% of all interventions), the obligatory drain removal was the most frequently performed intervention in the control group (8 | 12%). Looking specifically at the aspirations performed, almost 4 times as many aspirations were required in the TissuGlu^®^ group compared to the control group (mean number of aspirations 5.4, SD 3.4 vs. 1.7, SD 2.8; *p* = 0.014).

### Secondary endpoints

The group comparison showed that patients with a drain secreted approximately 12% more wound fluid than TissuGlu^®^ patients (total volume 578 ml, SD 393 vs. 514 ml, SD 420 ml; *p* = 0.779).

According to the protocol definition, all breasts treated with TissuGlu^®^ developed a seroma (100%), which had to be punctured at least once (Fig. 2). Only 63% (5/8) in the control group developed a seroma (Table 3).

**Table 3:** Secondary target parameters

Secondary endpoints	TissuGlu number | percentage respective mean (SD)	Control number | percentage respective mean (SD)
Output		
Drainage in domo [ml]	–	293 (174)
Drainage ex domo [ml]	–	145 (169)
Aspiration [ml]	514 (420)	139 (311)
Total volume [ml]	514 (420)	577 (393)
Wound conditions		
Seroma formation	8 | 100%	5 | 63%
Aspirations	5.4 (3.4)	1.7 (2.8)
Mean seroma size [cm]	1.5 (0.5)	1.7 (0.6)
Infection	2 | 25%	0 | 0%
Haematoma	2 | 25%	0 | 0%
Wound dehiscence	2 | 25%	0 | 0%
Time information [days]		
Days until seroma development	8.5 (3.9)	19.8 (5.1)
Drain in situ	–	12.6 (6.0)
Duration of in-patient stay	3.5 (0.7)	5.2 (3.3)
Days until adjuvant treatment	37.7 (14.4)	36.0 (10.9)
Unscheduled Visits (mean)	3.9 (3.14)	1.3 (1.60)
Number of unscheduled visits (Σ)	31 | 77.5%	9 | 22.5%

**Fig. 3:**
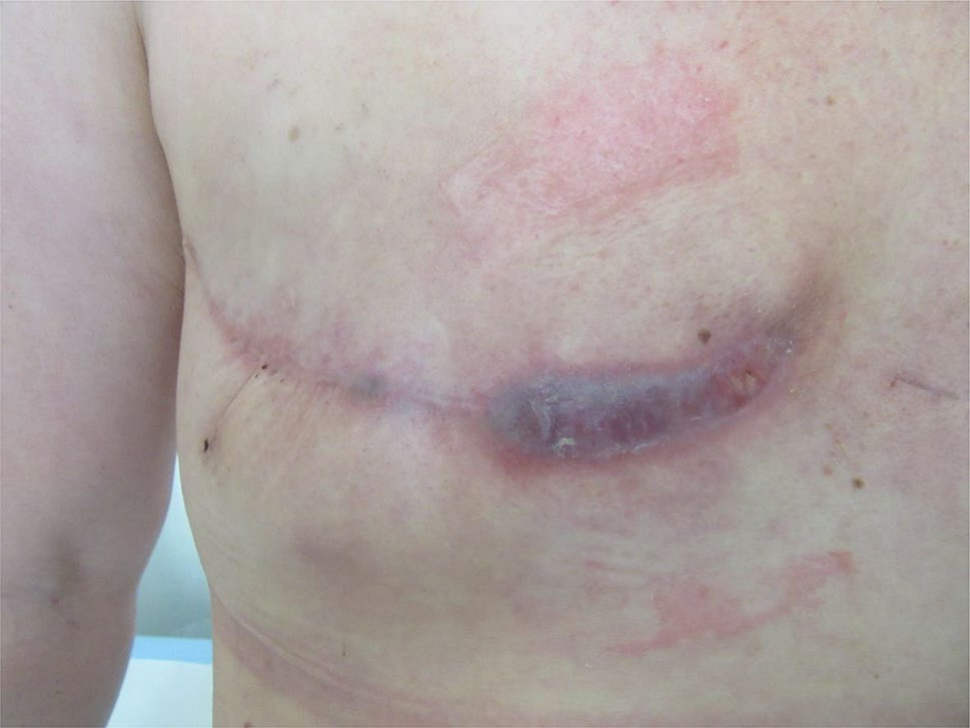
Illustration of wound dehiscence in a patient from the TissuGlu^®^ group with recurrent wound infections and consecutive revision after the end of the follow-up period

More wound complications were documented in the TissuGlu^®^ group: Infection, a haematoma or wound dehiscence were found in 25% of the patients (Fig. 3 and Table 3).

Two patients in the TissuGlu^®^ group had to undergo later revision with secondary sutures 4 and 5 months after surgery because of wound healing disorders, whereas in the control group there were no short- or long-term wound healing disorders.

A seroma was found significantly later in the control group, (8.5 days, SD 3.9 vs. 19.8 days, SD 5.1; *p* = 0.002); these were also less frequent (71.4% vs. 100%) than in the TissuGlu^®^ group, but this difference was not significant (*p* = 0.200).

The average duration of hospitalisation in the TissuGlu^®^ group was shorter (3.5 days, SD 0.8 vs. 5.2 days, SD 3.3; *p* = 0.662) and the number of unplanned post-operative presentations increased (3.9, SD 3.1 vs. 1.3, SD 1.6, *p* = 0.121), but not significantly.

There was no delay in subsequent adjuvant treatment in either group.

There were differences between the two groups with regard to postoperative pain, but these were not significant (Table 4).

**Table 4:** Pain survey during follow-up

Time	TissuGlu respective mean (SD)	Control respective mean (SD)
DFU1	12.9 (11.9)	28.7 (27.4)
DFU2	11.9 (9.7)	23.4 (33.9)
DFU3	14.3 (13.5)	20.0 (28.1)
FU07	14.9 (16.9)	6.5 (3.6)
FU14	13.2 (10.6)	8.0 (10.9)
FU30	10.4 (12.3)	4.2 (5.7)
FU60	1.6 (2.6)	8.9 (15.0)
FU90	1.3 (1.9)	5.4 (13.5)

Patients in the TissuGlu^®^ group reported less pain during the first days after surgery. A regression model with the factors time and group was formulated to describe the pain over the course of the study. The patients were considered as a random factor. In this model, the sensation of pain falls significantly with time (*p *= 0.013), whereas the group is not a significant factor (*p* = 0.403). The conditional pseudo *R*^2^ is 0.60, the marginal 0.12.

Patients in the TissuGlu^®^ group experienced less sleep disturbance and less reduced mobility. All patients in the drain group found that the drain device had a disturbing influence on sleep and 5 out of 6 (83%) stated that pain at the insertion site affected their sleep. Restricted mobility due to pain around the drain insertion site was reported by 33% (2/6); 5 out of 6 (83%) reported restricted mobility due to the drainage device.

## Discussion

The frequent occurrence of seromas is a problem for the surgical treatment of breast cancer therapy and its further treatment [2]. Wound drainage is the current standard, but it can be associated with side effects such as pain or restricted mobility.

The lysine-urethane-based tissue adhesive TissuGlu^®^ showed encouraging results in previous case reports, cohort comparisons and non-randomized studies in the field of senology, which are reviewed in this study [11–14].

Twice as many clinical interventions were performed in the TissuGlu^®^ group than in the drain group, especially aspirations of clinically relevant seromas (*p* = 0.014). Thus, 50% more aspirations were required in the test group than in the drain group (22 vs. 11 aspirations).

The only prospective trial so far has been published 2020 by Ohlinger et al. [16]. In his randomized controlled trial with 35 cases each, significantly more aspirations had to be performed in the TissuGlu^®^ group without drains; however, the number of total interventions did not differ significantly (*p* = 0.408). Very similar results were published by Ohlinger 2018 in his retrospective study [17].

In both studies a higher cumulative wound secretion was found in the drain group, although it was only significant in the retrospective study 2018. A similar tendency was observed in the present study (total volume 578 ml, SD 393 ml vs. 514 ml, SD 420 ml). Sauter et al. (2017) [18] also detected a significantly lower total secretion production in the TissuGlu^®^ group without drains, but there was no significant group difference in seroma formation or number of aspirations. A criticism of the study by Sauter et al. is that no randomization took place.

It is postulated that drains may provide a mechanical stimulus for increased secretion [19, 20]. To our knowledge there is no prospective study comparing two groups without drainage ± TissuGlu^®^.

In a study by Eichler et al. [21], 32 mastectomy patients with TissuGlu^®^ were compared with a retrospective cohort of 172 patients undergoing pure mastectomy. Both groups received drainage intraoperatively. No significant differences in seroma formation were found. However, the drain in the TissuGlu^®^ group could be removed much earlier (4.2 days vs. 3.5 days, *p* < 0.05). Thus, in this study the cumulative wound secretion was reduced by TissuGlu^®^.

This approach needs further evaluation in a prospective setting as it compares groups with the same characteristics minus the adhesive.

The postulated wound complications with drains [7] could not be reproduced. In the present evaluation, wound healing disorders such as infection and wound dehiscence only occurred in the TissuGlu^®^ arm. Although there was no significant difference in the overall analysis of all postoperative complications (*p* = 0.091) in the current study by Ohlinger 2020, wound dehiscence occurred only in the TissuGlu^**®**^ arm in 12.5% (*p* = 0.002) [16].

The reasons for this could be the more frequent aspirations or the trend towards higher BMI in the TissuGlu^®^ group (28.9 kg/m^2^, SD 4.9 vs. 24.4 kg/m^2^, SD 2.3 *p* = 0.108. Similarly, the results from Ohlinger [17] also showed significantly fewer seroma aspirations (*p* = 0.024) and complications (*p* = 0.012) in the control group. They also found a significant correlation (*p* = 0.030) between the BMI and the probability of the occurrence of a seroma in both groups—the higher the BMI, the greater the probability of a postoperative seroma.

Regarding quality of life, the results of the study by Findik et al. could be confirmed [6]: The patients in the TissuGlu^®^ group experienced less postoperative pain, less sleep disturbance and less reduced mobility. In questionnaires as well as in clinical observation, the lack of drain device, insertion site and the existing negative pressure in the tissue due to drainage suction proved to be an advantage.

**Fig. 4:**
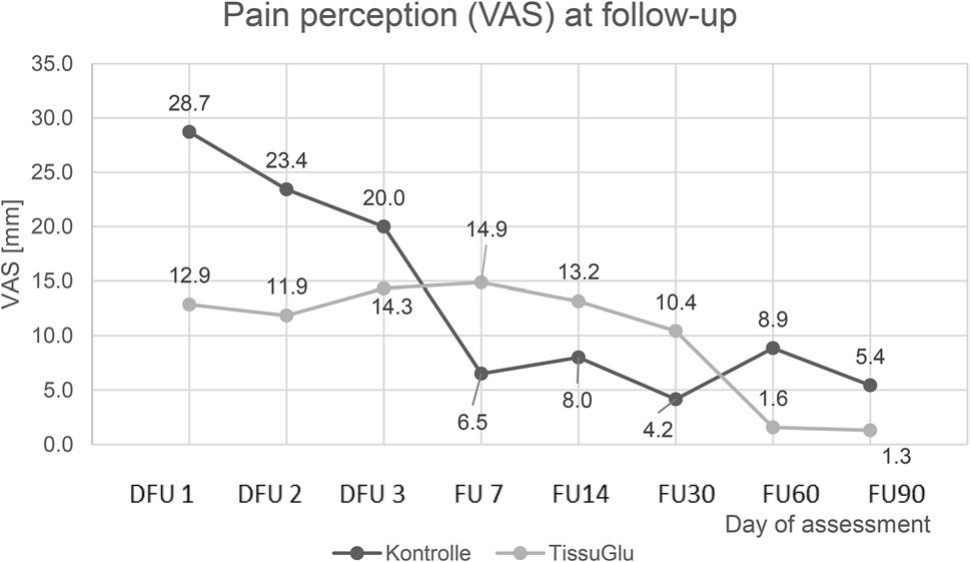
Pain perception during follow-up

In contrast to Ohlinger et al. [16] the evaluation of postoperative pain showed no significant difference between the two groups with the small group sizes: in both groups, pain after surgery decreased significantly with time (Fig. 4).

The TissuGlu^®^ group’s shorter inpatient stay (3.5 days vs. 5.2 days) certainly had a positive effect on the quality of life—in line with the previously cited studies—but at the cost of more frequent follow-up visits for aspiration.

The small number of cases resulting from early termination of the study by the study initiator because of slow patient recruitment is certainly the greatest limitation of the present study. Due to the small cohort, a potential relationship between patient-specific risk factors and the measurements taken could not be evaluated.

The evaluation of the available data and the clinical observations are consistent with figures from the available literature. For example, although TissuGlu^®^ appears to achieve reduced wound secretion by glueing the ventral and dorsal tissue surfaces together, seroma production does not decrease, therefore the purpose of application appears questionable. Until the study was discontinued, twice as many clinical interventions were performed in the TissuGlu^®^ group.

Furthermore, it remains to be discussed whether—as intended in the original study protocol—drain removal should be considered equivalent to aspiration.

Shorter hospitalisation times with potential savings and a higher quality of life due to the lack of drain insertion must be compared to the acquisition costs of the product (approx. 400 Euro) and more frequent aspirations with a higher seroma rate. In addition, more frequent outpatient follow-up was necessary, which also modifies the economic advantage.

There is evidence that TissuGlu^®^ leads to long-term sonographic tissue changes: an observational study 43 of 51 patients with intraoperative use of TissuGlu^®^

showed sonographic tissue changes in the following months, which mostly led to further histological clarifications [22].

It is doubtful whether data from abdominoplasty operations can be compared with chest surgery in the thoracic wall area. Compared to mastectomy, abdominoplasty is expected to result in similar tissue adhesion, but with minimal traumatisation of the lymphatic system.

In the field of reconstructive breast surgery as an interface between plastic and oncological procedures, a further study is currently being planned on the use of TissuGlu^®^ in breast surgery with autologous abdominal tissue [23].

This three-armed study was designed and initiated at the Cleveland Clinic (USA) in May 2019 with 198 patients. It remains to be seen whether tissue adhesive in the abdominal lift region will reduce the seroma rate.

## Conclusion

The present evaluation shows no advantage of the tissue adhesive TissuGlu^®^ in terms of seroma formation and frequency of intervention compared to an invasive drainage system for mastectomies, but the shorter inpatient stay certainly has a positive effect on the quality of life.

However, a completed prospective-randomized study with sufficient statistical power is necessary to make more definitive statements about the use of lysine-urethane-based tissue adhesives. Therefore, taking these results into account, TissuGlu^®^ should only be used in certain individual cases.
